# Does Biological Denitrification Inhibition (BDI) in the Field Induce an Increase in Plant Growth and Nutrition in *Apium graveolens* L. Grown for a Long Period?

**DOI:** 10.3390/microorganisms8081204

**Published:** 2020-08-07

**Authors:** William Galland, Florence Piola, Céline Mathieu, Lyna Bouladra, Laurent Simon, Feth el Zahar Haichar

**Affiliations:** 1 Université de Lyon, UMR 5557 LEM, Université Lyon 1, CNRS, INRA 1418, F-69622 Villeurbanne CEDEX, France; galland.william@outlook.fr (W.G.); lyna.bouladra@yahoo.fr (L.B.); 2 Université de Lyon, UMR5023 LEHNA, Université Lyon 1, CNRS, ENTPE, F-69622 Villeurbanne CEDEX, France; piola@univ-lyon1.fr (F.P.); laurent.simon@univ-lyon1.fr (L.S.); 3 Station d’Expérimentation Rhône-Alpes Information Légumes (SERAIL), 123 Chemin du Finday Les Hoteaux, 69126 Brindas, France; mathieu.serail@orange.fr; 4 INSA-Lyon, Université Claude Bernard Lyon1, CNRS, UMR5240, Microbiologie, Adaptation, Pathogénie, Univ Lyon, 10 Rue Raphaël Dubois, 69622 Villeurbanne, France

**Keywords:** biological denitrification inhibition (BDI), celery, fertilizers, procyanidins, nitrogen, plant traits

## Abstract

Intensive agriculture uses a lot of nitrogen fertilizers to increase crop productivity. These crops are in competition with soil-denitrifying microorganisms that assimilate nitrogen in the form of nitrate and transform it into N_2_O, a greenhouse gas, or N_2_. However, certain plant species exude secondary metabolites, called procyanidins, which inhibit denitrifiers and increase the nitrate pool in the soil available for plant nutrition. This phenomenon is called biological denitrification inhibition. Previously, we showed that the addition of exogenous procyanidins to a lettuce crop induces denitrifier inhibition and increases nitrate content in the soil, affecting lettuce morphological traits. Here, the effects of procyanidin amendments in the field on a more long-term and nitrogen-consuming crop species such as celery were tested. The effects of procyanidin amendment on celery growth with those of conventional ammonium nitrate amendments were, therefore, compared. Denitrification activity, nitrate concentration, the abundance of denitrifying bacteria in the soil, and traits related to celery growth were measured. It was shown that the addition of procyanidins inhibits denitrifiers and increases the soil nitrate level, inducing an improvement in celery morphological traits. In addition, procyanidin amendment induces the lowest nitrogen concentration in tissues and reduces N_2_O emissions.

## 1. Introduction

In the world, agricultural crops are very numerous and diversified [1], and there are several types of crops such as field crops [2,3], vegetable crops [4], and those involving arboriculture [5]. The plants of these crops do not present the same biological cycle and do not have the same nutrient requirements [6,7]. The current state of agriculture involves pushing for a significant increase in yields to meet increased consumer demand [8,9] and the demographic development of human populations on Earth [10].

Plants need nitrogen, which is one of the most important but also the most limiting factors for plant growth [11]. Plants assimilate nitrogen mainly in the form of ammonium (NH_4_^+^) but also as nitrate (NO_3_^−^) [12]. However, intensive agriculture requires the use of soils that may lack nutrients because of nitrogen depletion due to many successive crops [13,14] and direct competition with soil microorganisms for nitrate [15]. Among these microorganisms, archaea [16], fungi [17], and especially bacteria [18] transform NO_3_^−^ into N_2_ and N_2_O, the latter being a greenhouse gas, by denitrification processes [19]. Denitrification can result in a loss of up to 59% nitrogen in agricultural systems [20]. In addition, nitrate can also leach out of the soil [21] and potentially cause pollution or even human health problems [22].

To enhance crop yields, farmers amend soils with nutrients such as nitrogen fertilizers [23]. However, when applied, nitrogen fertilizers are not totally allocated to plants; these inputs are lost via different mechanisms, as mentioned above [24].

Nevertheless, under natural conditions, certain plant species, such as the invasive plant *Fallopia* spp., exude secondary metabolites called procyanidins that inhibit microbial denitrification activity, a phenomenon called biological denitrification inhibition (BDI) [25]. This phenomenon allows plants to limit the nitrate consumption of denitrifiers and, hence, allows plants to use the nitrate for their own nutrition and growth. Procyanidins are tannic molecules highly represented in the plant world, derived from the metabolic pathway of anthocyanins: compounds omnipresent in the secondary metabolism of plants [26].

Recently, Galland et al. [27] demonstrated that an exogenous supply of procyanidins in a field experiment involving lettuce crops induced BDI, which resulted in (i) a decrease in denitrification activity, (ii) an increase in nitrate concentration in plant tissues, and (iii) a significant increase in lettuce mass, compared to lettuces not amended with procyanidins. Moreover, lettuce (*Lactuca sativa*) tested by Galland et al. [27] is a crop that lasts only a few weeks and requires only one application of fertilizers [28] to allow the crop to reach term at acceptable and marketable yields. Other plants species such as celery (*Apium graveolens* L.) have a longer growing cycle than lettuce and require more nitrogen for their growth. In this case, farmers apply two important fertilizer inputs during cultivation [29]. In addition, celery can accumulate high nitrate concentrations in its tissues and, thus, a soil highly amended with nitrogen fertilizers could increase the concentration of nitrate within celery, causing health problems for the consumers [30,31]. Indeed, although useful and important at low doses for various functions of the human body [32,33], over-consumption via tissues can cause diseases such as methemoglobinemia [34] and the possibility of causing an endogenous formation of carcinogenic *N*-nitroso compounds [35]; it is, therefore, important for human health to monitor nitrate levels in consumed tissues.

In this study, we aimed to test the hypothesis that procyanidin amendment in a celery crop field experiment could provide nitrate supply for such a crop and reduce the use of fertilizer. To do so, we performed tests on an experimental field at the SERAIL experimental station (Brindas, 69126 Rhône, France), using plots planted with celery and amended with only one application of fertilizers and on others amended with the two conventional applications of fertilizers. Commercial procyanidins were amended at the same time as the fertilizer amendments, i.e., one time or two times on the planted plots. Unplanted plots and those planted with celery but not amended with either fertilizers or procyanidins were used as controls. Procyanidin concentrations were selected from the most effective concentration evidenced by Galland et al. [27], which was approximately 210 kg·ha^−1^ of procyanidins.

## 2. Materials and Methods

### 2.1. Plant Growth and Experimental Design

**Figure 1:**
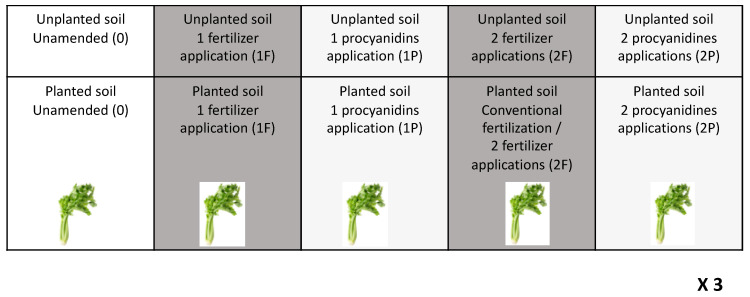
Summary diagram of the experimental design. Unamended (0), one fertilizer application (1F), two fertilizer applications (2F), one procyanidin application (1P), and two procyanidin applications (2P), for unplanted and planted plots, with a replicate number of three (× 3), following a Fisher model.

Celery (*Apium graveolens* L. var TANGO, Bejo) was grown on one field at the SERAIL experimental station (SERAIL, 69126 Rhône, Brindas, France) (45°43′46.4” north (N) 4°43′37.1” east (E)) composed of a sandy clay loam soil (sand: 68.7%, silt: 21.9%, and clay: 9.5% CEC: 58 meq·kg^−1^; organic carbon: 6.9 g·kg^−1^). The celery was planted in four parallel rows of 20 celery on 6 m × 1.4 m (8.4 m^2^) plots, with an unplanted continuity of 2 m × 1.4 m (2.8 m^2^) to measure the corresponding unplanted characteristics, following a Fisher system of three plots per treatment [36]. For soils planted with celery and the continuity of unplanted plots, five treatments were considered: unamended (0), amended once (1P) and amended twice (2P) with procyanidins, and amended once with fertilizer (1F) and amended two times with fertilizer (2F) (Figure 1). Celery was planted in the first week of June 2018. The first amendment with procyanidins at 210 kg·ha^−1^ was done on the eighth week of culture and, at the same time, the ammo-nitrate fertilizers were applied to adjust each plot to a concentration about 100 kg of N·ha^−1^. Plots 2P received, after the first application of 210 kg·ha^−1^ of procyanidins in week 8, a second application of 210 kg·ha^−1^ of procyanidins on the 11th week of culture and, at the same time, the 2F plots received the second fertilizer amendment of about 60 kg N·ha^−1^ of ammo-nitrate. The experiment was watered the first week after planting with 3 mm of water every day. For the next 10 days, 8 mm of watering was done per day. From that and until the end of the experiment, 12 mm of watering was done every two days. The commercial procyanidins (Laffort TANIN VR GRAPE ®112, Bordeaux, France; composed of 96% procyanidins C_30_H_26_O_12_ and the remainder composed of non-nitrogenous manufacturing residues) were applied in aqueous solution (standard water) by two nozzle spray booms, such as to give 500 L·ha^−1^ or 0.42 L per plot, between the rows of celery, and the soil was then hoed. Each site was hoed and was watered (with 8 mm) just after the addition of procyanidins. The results of the experiment were taken after 19 weeks of plant growth.

### 2.2. Denitrification Enzyme Activity (DEA)

As described by Galland et al. [27], denitrification enzyme activity (DEA), corresponding to the potential release of N_2_O expressed as μg N-N_2_O h^−1^·g^−1^ dry soil·h^−1^, was measured from 5 g dry equivalent (eq.) of pooled root-adhering soil (RAS) retrieved from four celery plants per plot and from four samples of bulk soil of unplanted plots, amended or unamended with procyanidins or fertilizers. BDI (%) was measured by calculating the percentage of denitrification activity inhibition recorded by DEA compared to the corresponding unamended control.

### 2.3. Quantification of Total Bacteria and Denitrifier Abundance

Total DNA was extracted from 0.5 g of RAS for six treatments (unplanted soil, RAS from unamended celery, celery with one and two fertilizer amendments, and celery with one and two procyanidin amendments), in triplicate, according to the manufacturer′s protocol of the Fast DNA Spin Kit for Soil (MP Biomedical, Solon, OH, USA). The amount of DNA extracted was then estimated using the Quant-iT PicoGreen® double-stranded DNA (dsDNA) Assay kit (Molecular Probes, Carlsbad, CA 92008, USA). Total bacteria and denitrifier abundances were quantified by quantitative PCR using primers targeting the 16S ribosomal RNA (rRNA) and *nirK*/*nirS* genes, respectively, as described previously by Galland et al. [27].

### 2.4. Nitrate Concentrations in Soil

For all plots, NO_3_^−^ was extracted one week before procyanidin/fertilizer application and on the day of harvest from the planted rhizospheric soils (20 cm from the base of four celery plants per plot and pooled) and from the unplanted soils. Nitrate was extracted from 5 g eq. of dried soil according to Galland et al. [27]. Briefly, nitrate was extracted from 5 g eq. of dried soil supplemented with 20 mL of a solution with 0.01 M CaCl_2_. Briefly, soil suspensions were shaken at 140 rpm for 2 h at 10 °C. The suspension was filtered (0.22 μm), and the NO_3_^−^ concentration was quantified using an ionic chromatograph ICS-900 (Thermo Scientific Dionex, Sunnyvale, CA, USA).

### 2.5. Measurement of Celery Traits

After 19 weeks of growth, for 10 celery plants per plot (30 per treatment), the height of the celery was measured using a ruler, and the shoots were then harvested and weighed on a balance (±0.5 g). In addition, four root systems per plot (12 per treatment) were harvested, washed using distilled water, and weighed on a precision balance (±0.001 g); then, they were dried at 68 °C for 24 h and weighed again on the same balance in order to determine dry masses.

### 2.6. Plant N Content

Total N concentration was measured using an elemental analyzer (FlashEA 1112, Thermo Scientific, Waltham, MA, USA) from three leaves and three root systems per plot (nine per treatment), using 2 mg of ground root or leaf material [27]. Leaf and root N content was expressed as a percentage of N in leaf and root dry mass.

### 2.7. Colorimetric Measurement of Nitrogen Stress

Using the device Dualex™ (FORCE-A, Orsay, France), the chlorophyll surface contents (expressed in Dualex units, relative unit of the manufacturer), and the nitrogen status NBI values (nitrogen balance index, being the ratio of measured chlorophyll/anthocyanin values) were measured on the last third leaf of 10 celery plants per plot (30 per treatment).

### 2.8. Data and Statistical Analyses

The difference in celery morphological traits (*n* ≥ 30) between treatments was determined using an ANOVA (analysis of variance) followed by a post hoc Tukey honestly significant difference (HSD) test. Similarly, the normality (Shapiro test) and the homoskedasticity of variance (Fischer test) of all the variables were tested. The significance of microbiological traits and nitrate concentration (*n* ≤ 3) between treatments was non-parametrically tested with a Kruskal–Wallis test followed by a post hoc Nemenyi. In addition, the plot effect was tested and discarded on all traits (two-way ANOVA (*p*-value: 0.78) and mixed model). All analyses were done using R project software (v. 3.5.0) (R Foundation for Statistical Computing, Vienna, Austria).

## 3. Results

### 3.1. Biological Denitrification Inhibition

**Figure 2:**
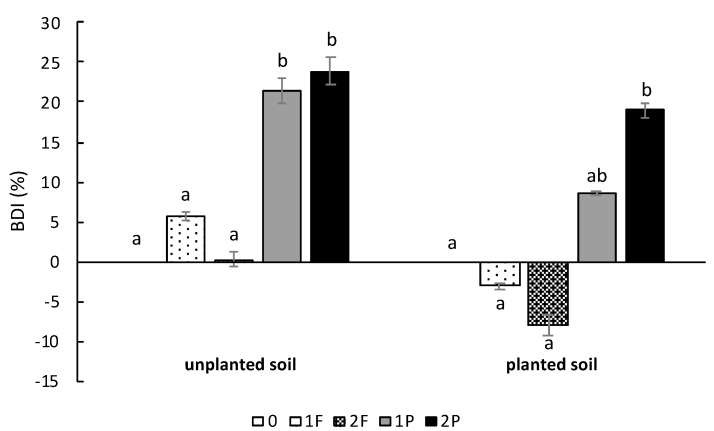
Impact of different field amendments on the percentage of biological denitrification inhibition (BDI). BDI (%) was calculated in relation to unamended soil for soil treatments and unamended plants for plant treatments. Unamended (0), one fertilizer application (1F), two fertilizer applications (2F), one procyanidin application (1P), and two procyanidin applications (2P). *n* = 4 for each treatment. Vertical bars indicate standard errors. Differences in letters (a, b and ab) indicate a difference in treatment (Nemenyi’s test; α < 0.05).

For the unplanted soil (Figure 2), the BDI was the same for the soils fertilized with one or two applications and the soil without amendment. However, the BDI of the unplanted soils amended with procyanidins was significantly lower (*p*-value: 0.048 for 1P and 0.025 for 2P) than that of the soils without amendment and amended with fertilizer.

For the planted soil (Figure 2), the BDI of soils amended with fertilizer once (1F) and twice (2F) was not significantly different from that of unamended soils (0). However, a tendency for denitrification stimulation was observed for the 1F and 2F plots. Notably, the addition of two doses of procyanidins induced a BDI of approximately 19%, which was significantly higher than that of the unamended soils (*p*-value: 0.023) and the soils amended with one (*p*-value: 0.019) or two (*p*-value: 0.017) fertilizer applications.

### 3.2. Abundance of Total and Denitrifying Bacterial Communities

**Figure 3:**
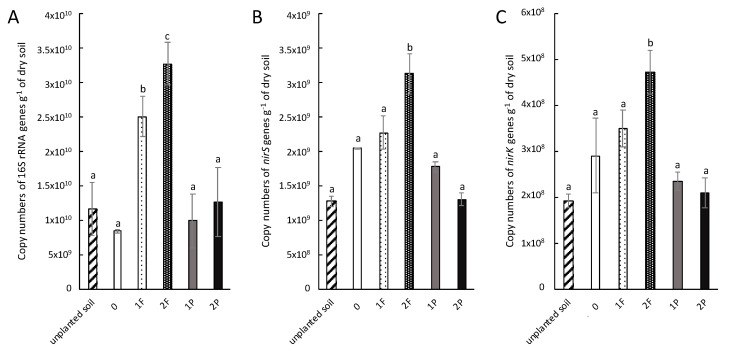
Amendment effects on total and denitrifying bacterial abundance. (**A**) Total bacterial community (copy numbers of 16S ribosomal RNA (rRNA)·g^−1^ of dry soil); (**B**) denitrifying bacteria with copy numbers of *nirK·*g^−1^ of dry soil; (**C**) denitrifying bacteria with copy numbers of *nirS·*g^−1^ of dry soil. Unplanted soil, unamended (0), one fertilizer application (1F), two fertilizer applications (2F), one procyanidin application (1P), and two procyanidin applications (2P). *n* = 3 for each treatment. Vertical bars indicate standard errors. Different letters (a, b and c) indicate which means differed (Nemenyi’s test, α < 0.05).

The abundance of the total bacterial community did not differ for unplanted soil, unamended celery, and celery amended with procyanidins (1P and 2P) (Figure 3A). However, the abundance was significantly higher in celery amended once and twice with fertilizer compared to unplanted soil, unamended celery, and celery amended with procyanidins (1P and 2P). The abundance of the *nirS* (Figure 3B) and *nirK* (Figure 3C) genes was not significantly different between unplanted and planted soil, with the exception of celery amended twice with fertilizer, for which abundance of the bacterial community harbouring *nirK* and *nirS* was significantly greater than that of the other treatments (Figure 3B,C). Interestingly, compared with unamended celery and celery amended with fertilizer, procyanidin applications tended to decrease the abundance of *nirS* and *nirK*. This trend was even more pronounced when procyanidins were applied twice.

### 3.3. Effects of Amendments on Soil Nitrate Content

**Figure 4:**
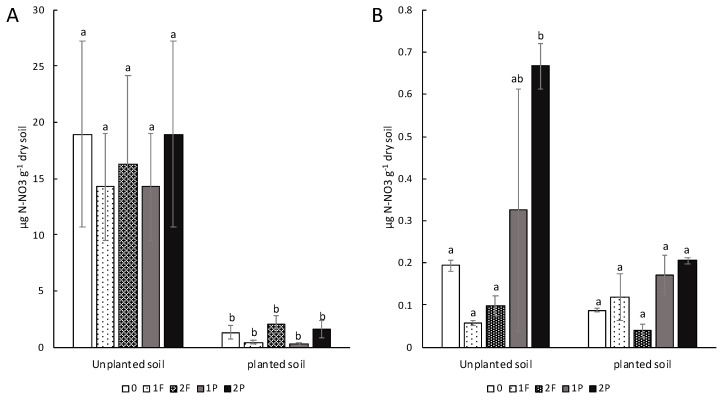
Effects of different types of amendments with procyanidins or fertilizers on soil nitrate level of unplanted soils and soils planted with celery: (**A**) for T0 before procyanidins or fertilizers amendments and (**B**) for TF corresponding to the day of harvest. Unamended (0), one fertilizer application (1F), two fertilizer applications (2F), one procyanidin application (1P), and two procyanidin applications (2P). *n* = 3 for each treatment. Vertical bars indicate standard errors. Differences in letters (a, b and ab) indicate a difference in treatment (Nemenyi’s test; α < 0.05).

One week before procyanidin application (T0) (Figure 4A), the nitrate level was the same for the unplanted soil treatments and for the celery treatments. However, significantly lower levels of nitrate were detected in the celery treatments compared to the unplanted soil treatments (*p*-value: 0.02). At harvest (TF), nitrate concentration in unplanted soil amended twice with procyanidin was significantly more important (*p*-value: 0.01) than that in the other treatments (Figure 4B). No significant differences were detected between the celery treatments (Figure 4B).

### 3.4. Effect of Amendments on Celery Traits

**Figure 5:**
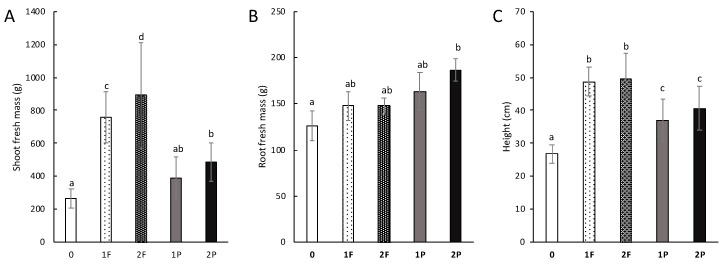
Impact of different field amendments on celery morphological traits. (**A**) The fresh mass of the celery aerial parts (g); (**B**) the fresh mass of the celery root systems (g); (**C**) the height of the celery aerial parts (cm). Unamended (0), one fertilizer application (1F), two fertilizer applications (2F), one procyanidin application (1P), and two procyanidin applications (2P). *n* = 30 for aerial parts and *n* = 6 for roots. Vertical bars indicate standard errors. Differences in letters (a, b, c and ab) indicate a difference in treatment (Tukey’s test for shoot fresh mass and height; Nemenyi’s test for root fresh mass; α < 0.05).

To test the effects of procyanidin and fertilizer amendments on celery traits, the shoot and root fresh mass and plant height were measured (Figure 5). The shoot fresh mass of celery grown on soils amended once and twice with fertilizer was significantly higher than that of the unamended celery and celery amended once and twice with procyanidins. The shoot fresh mass of celery amended twice with procyanidins had a shoot fresh mass of 487.4 g, representing a significant increase of approximately 222 g (83%) compared to that of unamended celery (*p*-value: 3.3 × 10^−5^). Interestingly, the fresh mass of the root system of celery amended twice with procyanidins was approximately 186.6 g, which was significantly higher by 60.2 g (or 47%) (*p*-value: 0.024) than that of unamended celery (Figure 5B). Notably, although not significant, the fresh root masses of celery amended with procyanidins tended to be 39 g higher on average than the fresh mass of celery amended with fertilizer. Celery amended once with procyanidins had an average height of 36.9 cm, which was significantly higher by 10.2 cm on average (or 37%) than that of the unamended celery (*p*-value: 1.33 × 10^−6^). The height of celery amended once with procyanidins was statistically no different from that of celery amended twice. The height of celery treated with two procyanidin amendments was significantly higher by an average of 13.8 cm (or 52%) than that of the unamended celery (*p*-value: 4 × 10^−8^). Notably, the height of celery amended once or twice with fertilizers was significantly higher than that of unamended celery and celery amended once or twice with procyanidins (Figure 5C).

### 3.5. Effect of Amendments on Plant N Content

**Figure 6:**
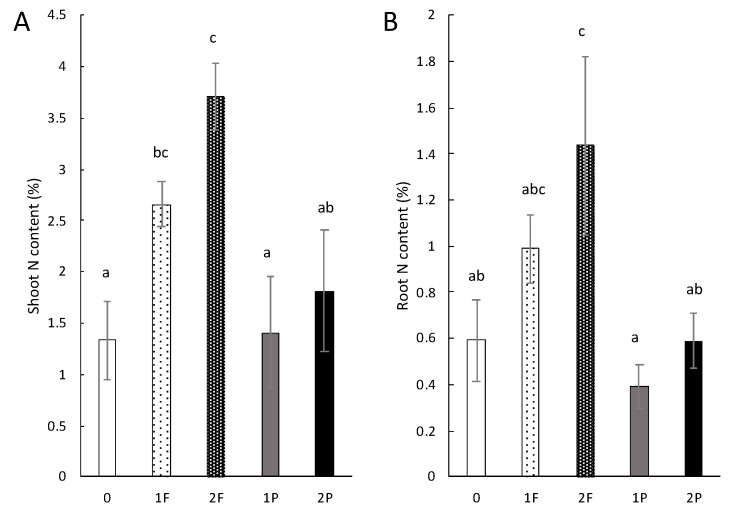
Amendment effects on tissue N content. (**A**) Shoot and (**B**) root N content (% of dry mass). Unamended (0), one fertilizer application (1F), two fertilizer applications (2F), one procyanidin application (1P), and two procyanidin applications (2P). *n* = 3 for each treatment. Vertical bars indicate standard errors. Differences in letters (a, c, ab, bc, and abc) indicate a difference in treatment (Nemenyi’s test; α < 0.05).

The percentage of nitrogen content in the shoots of unamended celery was, on average, approximately 1.3%. The N content was not affected by one procyanidin application, as we obtained the same level as that in the unamended celery (Figure 6A). However, the percentage of N in the leaves of celery amended once and twice with fertilizer was approximately 2.66% and 3.7%, respectively, which was significantly higher than the N content of the shoots of unamended celery and those of celery amended once with procyanidins. In addition, the N percentage of shoots of celery amended twice with fertilizer differed significantly from that of celery amended twice with procyanidins.

The N content in unamended celery roots was approximately 0.59% and did not differ significantly from that of celery amended once (0.39%) and twice with procyanidins (0.59%) (Figure 6B). The N concentration in the roots of celery amended twice with fertilizer was approximately 1.4% and tended to be higher than that of celery amended once with fertilizer, albeit not significantly. This concentration was significantly higher than that of unamended celery (*p*-value: 0.04), as well as celery amended once (*p*-value: 0.01) and twice with procyanidins (*p*-value: 0.04).

### 3.6. Effect of Amendments on Colorimetric Measurements of N Stress

**Figure 7:**
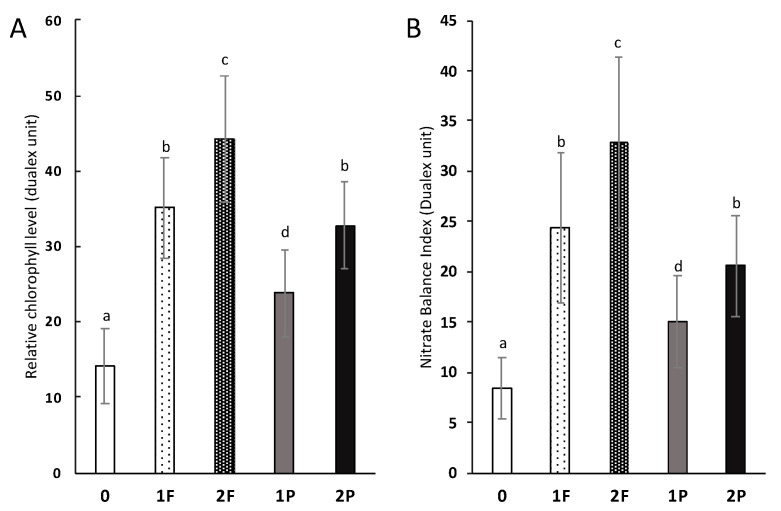
Impact of different field amendments on the pigment level of celery leaves. (**A**) Relative chlorophyll level (Dualex unit) and (**B**) nitrate balance index (Dualex unit) of the last third celery leaf. Unamended (0), one fertilizer application (1F), two fertilizer applications (2F), one procyanidin application (1P), and two procyanidin applications (2P). *n* = 30. Differences in letters (a, b, c and d) indicate a difference in treatment (Tukey’s test; α < 0.05).

The relative chlorophyll content of the leaves of unamended celery was 14.2 Dualex units and was significantly lower than that from leaves of celery amended once (*p*-value: 4 × 10^−6^) and twice with procyanidins (*p*-value: 1 × 10^−7^) and once (*p*-value: 8 × 10^−8^) and twice with fertilizers (*p*-value: 3 × 10^−9^) (Figure 7A). The chlorophyll content of the leaves of celery amended twice with procyanidins did not differ from that of the leaves of celery amended once with fertilizer, but it was significantly lower than that of the leaves of celery amended twice with fertilizer (*p*-value: 6 × 10^−7^). The nitrogen balance index (NBI) measured in the leaves of unamended celery was significantly lower than that measured in the leaves of celery amended once (*p*-value: 0.03) and twice with procyanidins (*p*-value: 5 × 10^−^^7^), and once (*p*-value: 7 × 10^−^^7^) or twice (*p*-value: 8 × 10^−^^7^) with fertilizer. Notably, the NBI measured in the leaves of celery amended twice with procyanidins did not differ from that in the leaves of celery amended once with fertilizer (Figure 7B).

## 4. Discussion

### 4.1. Procyanidin Amendment to Celery Plots Induced an Increase in BDI and Changes in Denitrifiers Abundance

The addition of procyanidins at 210 kg·ha^−1^ in the field induced a BDI as previously observed in Galland et al. [27]. This BDI was more notable in the planted and unplanted plots amended twice with procyanidins. Two procyanidin amendments may increase procyanidin bioavailability in the soil, consequently inducing less microbial denitrification activity. The BDI tended to be lower when measured on planted soil than on unplanted soil with equivalent treatments. This result suggests that the BDI is not stimulated by plants. In contrast, probably via their rhizosphere (root exudation, root respiration, and nitrate availability), plants stimulate denitrification activity [37], thus decreasing the BDI. In addition, the BDI measured in soil planted with celery amended only one time with procyanidins and in unplanted soil tended to be lower than that in plots with celery amended twice with procyanidins, probably due to the resilience of denitrifiers in these plots or the improved bioavailability of procyanidins following two applications. In the plots amended only once with procyanidins, there were approximately 76 days between the last procyanidin amendment and the BDI measurements, while, in plots amended twice with procyanidins, there were only 55 days between the last amendment and the BDI measurements. It seems that a longer time between the moment of procyanidin application and BDI measurement results in a lower BDI. This result suggests that the effect of procyanidins on denitrifier activity decreases in a relatively short period of time, as described by Zerulla et al. [38], who showed that the level of nitrification activity returned to the initial nitrification activity level of soil bacterial communities at 4–10 weeks after the addition of 3,4-dimethylpyrazole phosphate (DMPP), a nitrification inhibitor, to the soil.

As shown in Galland et al. [27], procyanidin amendment did not affect the abundance of the total bacterial community, and it tended to reduce only the abundance of denitrifiers, which seems to be counter-selected by plants as they lost their function. Moreover, the abundance of denitrifying bacterial communities tended to be relatively low when there were two procyanidin applications rather than one, supporting the hypotheses suggested above that this is either an effect related to the increased bioavailability of procyanidins following two applications or a trend toward the resilience of denitrifying bacterial communities when procyanidins were applied once.

Planted soil fertilized once or twice with ammonium nitrate induced microbial denitrification activity and, thus, N_2_O emissions. This result is consistent with a previous study by Mulvaney et al. [13], who explained that N fertilizer promotes the denitrification process. Similarly, the abundance of denitrifying communities seems to be stimulated by fertilization in agreement with a study by Sun et al. [39] demonstrating that N fertilizer induces an increase in the abundance of communities involved in the N cycle, including those of denitrifiers. In addition, N fertilizer amendment induced an increase in the abundance of the total bacterial community, as shown in Reference [40]. Moreover, the addition of fertilizer resulted in an increase in the nitrate pool in the soil, stimulating denitrification activity and inducing both high N_2_O emissions and nitrate loss from the soil, as also observed in Venterea et al. [41].

### 4.2. The Addition of Procyanidins to Celery Plots Modifies the Soil Nitrate Concentration

Soil nitrate pool was increased in unplanted soils amended once and twice with procyanidins as observed by Bardon et al. [42]. No conservation of nitrate was observed in the planted soil, as celery plants consumed nitrate for their growth [43]. Interestingly, the nitrate concentrations in the unplanted and planted soils amended once or twice with fertilizer were lower than those in soils with celery amended with procyanidins. This finding could be explained by microbial denitrification activity, which was more important in the fertilized plots than in amended plots with procyanidins where denitrification activity was inhibited, allowing relatively more nitrate in those plots.

### 4.3. The Addition of Procyanidins to Celery Plots Improves Celery Growth Traits

The addition of procyanidins increased the fresh mass of the shoots and roots, as well the height of the celery plants. This increase was more important when the celery was amended twice with procyanidins, suggesting that this treatment made nitrate more available and for a relatively longer period than did the single amendment.

Compared to the shoots of unamended celery plants, there were gains of approximately 47% and 53% in the shoots of plants amended once or twice with procyanidins, respectively. These gains were very low compared with those of conventional celery amended twice with fertilizer (236%) and celery amended once with fertilizer (186%). Thus, treatment with fertilizer seems to be relatively effective but leads to increased levels of nitrate in the soil, likely consumed by denitrifiers, generating increased amounts of N_2_O emissions into the atmosphere. Thus, to potentially make more nitrate available to plants, it might be possible to increase the percentage of BDI by adding a third application of procyanidins to crops. This application would further inhibit the denitrifying microbial community, increasing the amount of nitrate available for plants and bringing their yields closer to conventional yields without releasing nitrate into agrosystems, which can negatively impact the environment.

In addition, celery amended with procyanidins developed more roots than shoots when amended with procyanidins than when amended with fertilizer. As the denitrifier distribution in the soil is heterogeneous, nitrates available to plants due to denitrifier inhibition would also be heterogeneously spatially distributed in the soil. To benefit from this nitrate, plants concentrate new and more root growth in the nitrate-containing zone, which could explain the gain in root mass, as previously demonstrated for young lettuce plants subjected to a heterogeneous spatial distribution of nitrate in the root zone [44].

In addition, N levels in the shoot and root tissues did not differ between unamended celery and celery amended once or twice with procyanidins, and they were lower than those obtained from celery amended once or twice with fertilizer, confirming the results of the van Wassenhove et al. [45] study, which showed that the application of nitrogen fertilizer to a celery crop increases the nitrate concentration in the leaves. Thus, the nitrate available following the addition of procyanidins was concentrated little in celery tissues, and the physiology of that celery did not differ from that of unamended celery. This relatively low nitrate concentration could mean a relatively low risk for consumer health [30,31].

In the same way, the results of the colorimetry measurements exhibited the same trends as those of the other measured features. According to Cerovic et al. [46], chlorophyll levels are relatively high when plants are not stressed by soil N levels, and the plants allocate relatively high amounts of resources to their primary metabolism. These values were lower in all plots than in those with conventionally amended celery. In addition, the NBI measured by this device, as described by Jezek et al. [47], provides information on the N status of the plant because an N-deficient plant will produce less chlorophyll and relatively more flavonoids than a nitrogen-sufficient plant, which will produce the opposite. Thus, the NBI allows us to compare between treatments to obtain more specific data on N-related stress. The NBI was higher when celery was amended with procyanidins that when celery was not amended, which indicates less stress on the plant, but this stress related to soil N was higher than when celery was amended with fertilizer. However, it is interesting to note that the NBI measured in celery amended once with fertilizer was similar to that measured in celery amended twice with procyanidins, which means that plant N stress tended to decrease under the double procyanidin treatment. This stress may decrease further if a third application of procyanidins is applied to a crop, which could also decrease the NBI and increase the chlorophyll levels, tending to make them equal to the levels of conventional celery.

## 5. Conclusions

The addition of procyanidins to celery fields induced a BDI, increased the nitrate pool available in the soil, and increased the morphological traits related to growth of celery compared to the unamended celery. These traits are less important than those obtained for celery amended with fertilizers in a conventional agriculture. For the first time, it was shown that N levels in the plant remain lower when celery was amended with procyanidins than fertilizers and potentially represents a lower risk of nitrate contamination for the consumers. Although the aerial masses of these celery amended with procyanidins are smaller than conventional celery, they are still large enough to be placed on the market. In order to be close to the masses of conventional celery without increasing N plant level, a third procyanidin amendment could be tested to induce more BDI and, hence, more nitrate available for plant growth. It would also be possible to couple a fertilizer amendment with a procyanidin amendment in order to limit the risks associated with excessive N input into the soil and in-fine into the plant, while limiting via procyanidin amendment the loss by denitrification of N input to the soil. It would be interesting in the future to take into account the more organic nature of this type of amendment, which did not have a negative effect on the environment by emitting N_2_O to the atmosphere as is the case for conventional celery production.
